# Differences in glucose metabolic activity in liver metastasis separates two groups of metastatic uveal melanoma patients with different prognosis

**DOI:** 10.1002/cam4.6058

**Published:** 2023-05-21

**Authors:** Luis P. del Carpio, María Asunción Algarra, Aida Sabaté‐Llobera, Alejo Rodriguez‐Vida, Susana Rossi‐Seoane, Sandra Ruiz, David Leiva, Emilio Ramos, Laura Lladò, Daniel Lorenzo, Cristina Gutierrez, Montserrat Cortes‐Romera, Josep M. Caminal, Josep M. Piulats

**Affiliations:** ^1^ Medical Oncology Department Institut Català d'Oncologia ‐ ICO, L'Hospitalet de Llobregat, IDIBELL Barcelona Spain; ^2^ Cancer ImmunoTherapy (CIT) Group – iPROCURE, Bellvitge Biomedical Research Institute IDIBELL‐OncoBell, L'Hospitalet de Llobregat Barcelona Spain; ^3^ Medical Oncology Department Instituto Valenciano de Oncología – IVO Valencia Spain; ^4^ Nuclear Medicine Department Institut de Diagnòstic per la Imatge ‐ IDI, Hospital Universitari de Bellvitge, L'Hospitalet de Llobregat Barcelona Spain; ^5^ Medical Oncology Department Hospital del Mar Barcelona Spain; ^6^ Nuclear Medicine Department Centro Uruguayo de Imagen Molecular – CUDIM Montevideo Uruguay; ^7^ Radiology Department Hospital de Bellvitge, L'Hospitalet de Llobregat Barcelona Spain; ^8^ Hepatic Surgery Department Hospital de Bellvitge, L'Hospitalet de Llobregat Barcelona Spain; ^9^ Ophthalmology Department Hospital de Bellvitge, L'Hospitalet de Llobregat Barcelona Spain; ^10^ Radiation Oncology Department Institut Català d'Oncologia ‐ ICO, L'Hospitalet de Llobregat Barcelona Spain; ^11^ Centro de Investigación Biomédica en Red de Cáncer – CIBERONC Madrid Spain

**Keywords:** glucose metabolism, liver metastasis, PET/CT, prognostic, uveal melanoma

## Abstract

**Background:**

Uveal melanoma metastasizes to the liver. We aimed to explore the metabolic activity of liver metastases (LM) as a biomarker for survival.

**Methods:**

We analyzed newly diagnosed patients with metastatic UM (MUM) with LM detected by liver‐directed imaging and had undergone a PET/CT at diagnosis.

**Findings:**

51 patients were identified between 2004 and 2019. Median age was 62 years, 41% male and 22% ECOG ≥1. LDH, ALP, and GGT were elevated in 49%, 37%, and 57% of patients. Median LM SUVmax was 8.5 (3–42.2). Same size lesions presented a wide range of metabolic activity. Median OS was 17.3 m (95% CI:10.6–23.9). Patients with SUVmax ≥8.5 had an OS of 9.4 m (95% CI:6.4–12.3), whereas patients with SUVmax <8.5 had an OS of 38.4 m (95% CI:21.4–55.5; *p* < 0.0001, HR = 2.9). We observed similar results when studying M1a disease separately. Multivariate analysis showed SUVmax as an independent prognostic factor for the whole population and those with M1a disease.

**Interpretation:**

Increased metabolic activity of LM seems to be an independent predictor of survival. MUM is a heterogeneous disease and metabolic activity probably reflects a different intrinsic behavior.

## 1 INTRODUCTION

Uveal melanoma (UM) is a rare disease but the most common primary intraocular malignant tumor in adults, with an annual incidence of six cases per million. Overall survival (OS) at 5 years ranges from 50% to 65%.^1^, ^2^, ^3^ This elevated mortality rate is caused by a high incidence of metastases that are usually fatal within 16–22 months after diagnosis.^4^, ^5^, ^6^, ^7^ The pattern of metastatic spread differs from cutaneous melanoma since the absence of lymphatic drainage in the eye leads UM to metastasize hematogenously, predominantly to the liver.

Several characteristics of the primary tumor such as ciliary body involvement; extrascleral extension; diameter and location of the anterior margin of the tumor; cell type; presence of mitotic figures; tumor‐infiltrating lymphocytes and macrophages; the presence of vascular loop and pigmentation; chromosome 3, 6p an 8q status; and class 1 or 2 gene expression profiles have been identified as prognostic factors for metastatic relapse.^8^, ^9^, ^10^ However, there continues to be a dearth of research providing a multivariate analysis of prognostic factors in the metastatic setting. Based on these studies, age, gender, interval of time between UM primary treatment and metastasis diagnosis, and factors related with the severity of liver metastatic burden; such as performance status, percentage of hepatic replacement, metastasis diameter, and abnormalities in liver function tests, have all been shown to have an impact on OS.^11^, ^12^, ^13^, ^14^ Despite all these studies, only the diameter of the largest metastasis has been considered robust enough to be included in the American Joint Committee on Cancer (AJCC) TNM classification system.^15^

[^18^F]‐fluorodeoxyglucose (FDG) positron emission tomography (PET) integrated with computed tomography (CT) combines functional information about glucose cell metabolism with the anatomic location and characteristics of the detected areas of FDG uptake. This technique is widely used to stage patients with cutaneous melanoma.^16^ The few studies that have been performed to determine its effectiveness in detecting primary UM tumors have demonstrated correlations between high glucose uptake and histological adverse prognostic factors.^17^, ^18^ In these same studies, FDG‐PET/CT detected low incidence of metastatic disease but high incidence of second primary malignancies.^19^, ^20^ Most of the few studies dealing with MUM simply compare the capacities of FDG‐PET/CT and magnetic resonance image (MRI) to diagnose liver metastasis (LM).^21^, ^22^ In all the studies, MRI showed superiority to FDG‐PET/CT mainly because of heterogeneity in glucose uptake.

The prognosis of patients with metastatic UM (MUM) can vary widely from months to years regardless of the treatment received.^11^, ^12^, ^13^ There remains a need for useful tools that enable medical oncologists to better determine patients' prognoses. These tools could additionally be used to select patients that are candidates for more aggressive liver‐directed therapies or stratify patients in clinical trials. In an attempt to develop this type of tool, we explore a new application of FDG‐PET/CT in the initial assessment of LM from UM to evaluate disease glucose metabolism as a biomarker for OS.

## 2 METHODS

### 2.1 Study design and participants

**FIGURE 1:**
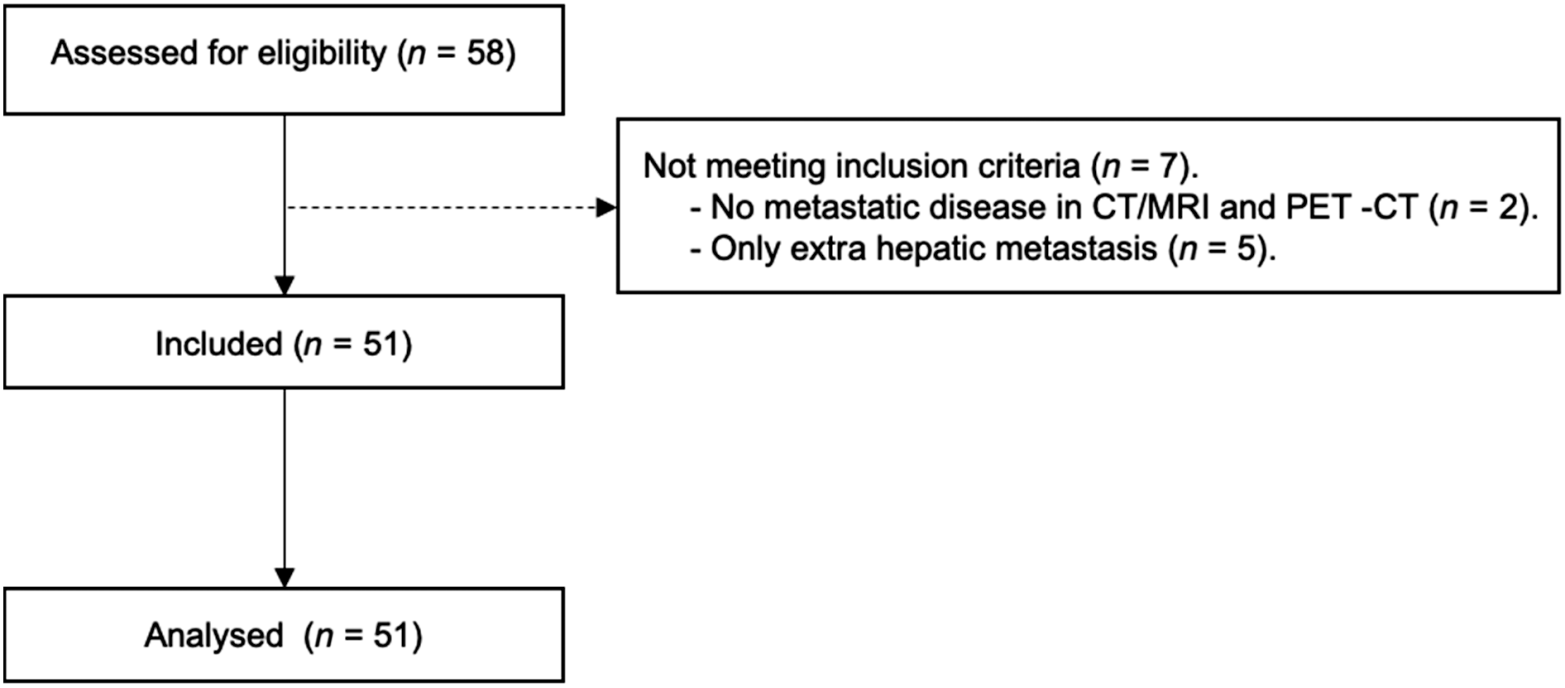
CONSORT diagram. CT, computerized tomography; MRI, magnetic resonance imaging; PET, positron emission tomography.

We retrospectively identified 58 MUM patients who had undergone a FDG‐PET/CT between June 2004 and September 2019. Study entry required a FDG‐PET/CT performed for newly diagnosed MUM, and a detectable metastasis on a liver‐directed imaging study (CT or MRI). A histologically proven liver biopsy was required for cases with LM identified by CT and/or MRI but without metabolic activity detected in the FDG‐PET/CT. Two patients were excluded because no metastasis was found on the FDG‐PET/CT and liver‐directed imaging with CT or MRI. Five patients with only extrahepatic metastatic disease were also excluded. Based on these criteria we identified and analyzed 51 patients. A CONSORT diagram is shown in Figure 1.

Patients' characteristics are described in Table 1. Median age was 62 years (range 30–84), 21 were male, 40 were asymptomatic based on the Eastern Cooperative Oncology Group‐Performance Status (ECOG‐PS). The levels of lactate dehydrogenase (LDH) and alkaline phosphatase (ALP) were normal in 26 and 32 patients, respectively. Median diameter of the largest LM was 20 mm (range 8–178) with 17 patients showing largest LM ≥30 mm. Up to 19 patients had additional extrahepatic metastatic involvement. Extrahepatic metastasis location and maximum standardized uptake value (SUVmax) for liver and extrahepatic lesion with the most FDG uptake are presented in Table S1.

**TABLE 1:** Characteristics of patients (data from *n* = 51).

Characteristics	Categories	Number (%; *N* = 51)
Sex	Male	30 (59)
	Female	21 (41)
Age, years (median 62, range 30–81)	<65	31 (61)
	≥ 65	20 (39)
ECOG/performance status	0	40 (78)
	≥ 1	11 (22)
LDH	Normal	26 (51)
	Elevated (greater than ULN)	25 (49)
ALP	Normal	32 (63)
	Elevated (greater than ULN)	19 (37)
GGT	Normal	22 (43)
	Elevated (greater than ULN)	29 (57)
Site of metastases	Hepatic alone	32 (63)
	Hepatic and extra‐hepatic	19 (37)
Diameter of largest metastases (mm)	<30	34 (67)
(median 20, range 8–178)	≥30	17 (33)
Metastases‐free survival (years)	<2	18 (35)
(median 2.7, range 0–20.8)	≥2	33 (65)

Abbreviations: ALP, alkaline phosphatase; ECOG, Eastern Cooperative Oncology Group; GGT, gamma‐glutamyltransferase; LDH, lactate dehydrogenase; ULN, upper limit of normal.

The Hospital de Bellvitge/Catalan Cancer Institute Institutional Review Boards and Ethics Committee at IDIBELL approved this study for clinical investigation. All the methods were conducted in conformity with the Declaration of Helsinki and relevant guidelines. No patients are alive at the time of this analysis.

### 2.2 Image acquisition and analysis

FDG‐PET/CT and liver‐directed imaging, either CT or MRI, were indicated at the moment LM were suspected by liver ultrasound, which is routinely performed during follow‐up. For the FDG‐PET/CT scan, patients were instructed to fast for at least 6 h and to drink water willingly. Plasma glucose levels at the time of FDG injection were required to be under 200 mg/dL. PET/CT, CT, and MRI images were reviewed by a nuclear medicine physician and a radiologist in dedicated workstations. For PET images, SUVmax was obtained for the lesion with the most intense FDG uptake, and the ratio between this value and FDG uptake in the healthy liver parenchyma was calculated. We determined SUVmax and analyzed it as a continuous variable, and also dichotomized it according to the obtained median SUVmax value (<median SUVmax vs. ≥median SUVmax). If no LM were identified by PET‐CT, those were categorized as <median SUVmax.

### 2.3 Statistical analysis

Categorical data were described as frequencies and percentages. Continuous variables were presented as medians and ranges. Chi‐square test or Fisher's exact test were used for comparisons between categorical variables. OS was calculated from date of diagnosis of metastatic disease to last control status. Survival curves were estimated using the Kaplan–Meier method. The log‐rank test was used to compare groups. The Cox proportional hazards model was used to estimate the hazard ratio (HR) with 95% confidence intervals (CI). Both univariate and multivariate models were used. SUVmax was used both as a continuous and dichotomous variable, according to median, for each model. Linear regression models were used to fit metastasis size and SUVmax value against FDG uptake in normal liver. In regard to the ratio between metastasis size and FDG uptake in normal liver, different regression models were used for ≤30 mm and for >30 mm metastases. Similarly, for the ratio between M1 SUVmax and FDG uptake in normal liver, different regression models were used for metastasis SUVmax ≤8.5 and >8.5. Beta coefficients and *p*‐values were calculated. *p*‐values of <0.05 were considered statistically significant. All statistical analyses were performed using R version 4.0.1.

## 3 RESULTS

### 3.1 Imaging findings

The median time between liver‐directed image with CT and/or MRI, and FDG‐PET/CT was 7.4 days with a maximum of 48 days. Overall, all 51 patients were thought to have liver disease according to CT and/or MRI. On the other hand, FDG‐PET/CT was positive in 45 of these 51 patients. FDG‐PET/CT was unable to detect LM in 6 patients.

Median SUVmax from hepatic lesion with maximum uptake was 8.5 (range 2.6–42.2). In order to explore differences between patients having liver lesions with a similar FDG uptake to normal hepatic parenchyma from those with a higher uptake, we determined the ratio between SUVmax in the highest FDG uptake lesion and the normal liver uptake in the same patient (FDG‐ratio). Ten of the 51 patients (19.6%) were considered iso‐metabolic because the FDG uptake was found to be similar to normal liver (FDG‐ratio < 1.1). The six patients with undetectable LM by FDG‐PET/CT were included in this group. The median diameter of the largest hepatic metastasis was 10.5 mm (range 8–50) in iso‐metabolic tumors, and 11 mm (range 8–15) when patients with undetectable LM by FDG‐PET/CT were analyzed separately. Among the 41 patients considered hyper‐metabolic, with higher FDG uptake (FDG‐ratio ≥ 1.1), the median diameter of the largest hepatic metastasis was 24 mm (range 10–178). Patient characteristics in both groups are shown in Table S2. There were no clinical, biochemical, or radiological differences found to be statistically significant between iso‐metabolic and hyper‐metabolic tumors.

**FIGURE 2:**
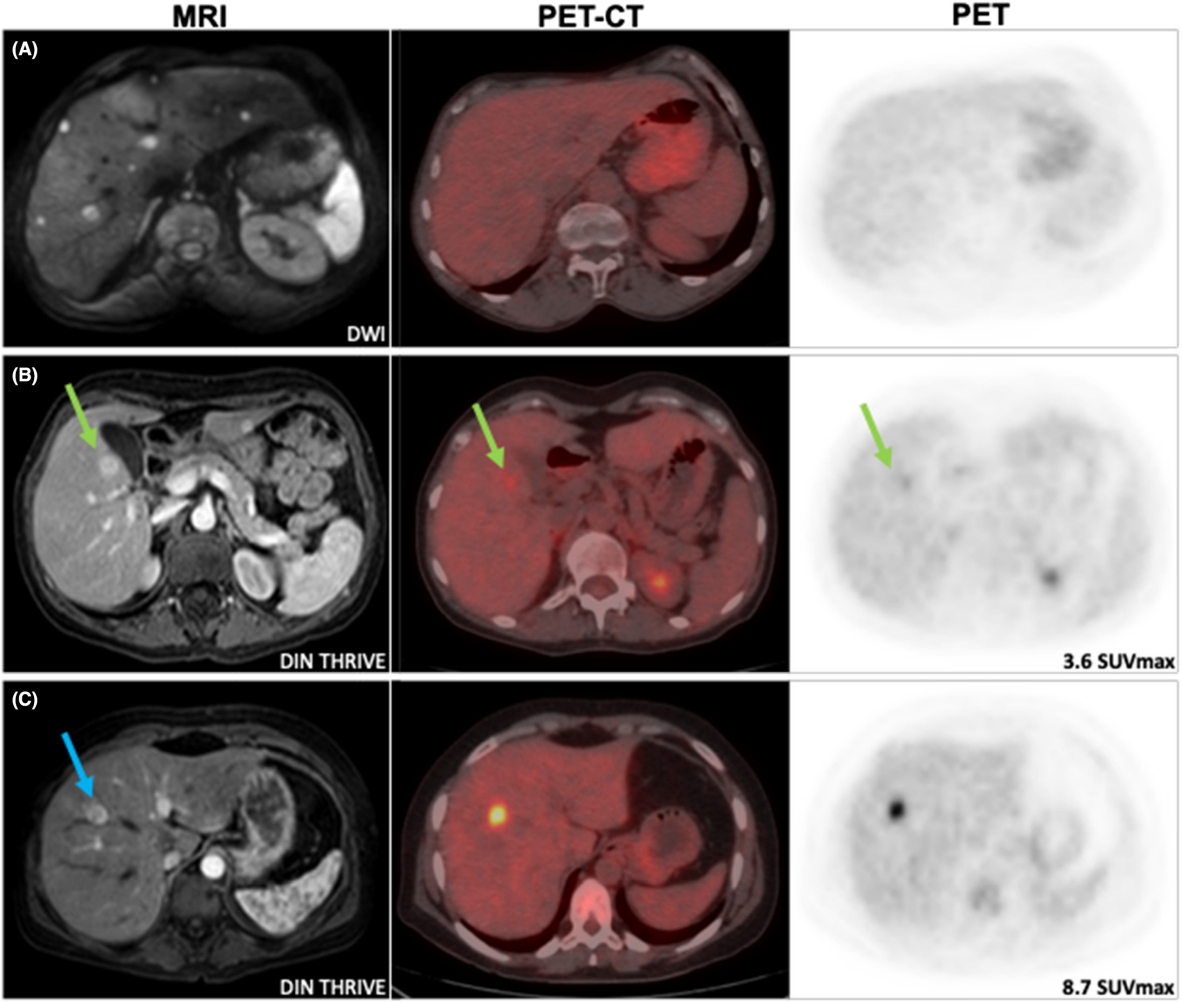
Different degree of FDG uptake in lesions of 15 mm. Lesions are clearly visible in conventional morphological imaging MRI though they can show no increase in FDG uptake with the same activity as healthy liver (A), mild hypermetabolism (green arrow; B), or more significant hypermetabolism (blue arrow; C). All three lesions are depicted as hyperintense in T2 and STIR with a restricted diffusion pattern, and hipervascular after gadolinium I.V. administration in arterial phase by MRI. Lesions are hypointense in T1, with the exception of lesion C that shows central hyperintensity in T1 suggesting melanin deposits in the tumor. CT, computerized tomography; DIN THRIVE, T1W high‐resolution isotropic volume examination; DWI, diffusion weighted imaging; MRI, magnetic resonance imaging; PET, positron emission tomography.

Lesions larger than 50 mm in size were always hypermetabolic (ratio ≥ 1.1), and lesions less than 10 mm in size were always iso‐metabolic (ratio < 1.1) compared with normal liver uptake. Interestingly, different FDG uptakes were observed among different patients with relatively similarly sized lesions (Figure 2; Figure S1). These discrepancies in FDG uptake among same size tumors indicate that tumor metabolism might be a prognostic factor for OS, regardless of metastases size in patients with MUM with liver involvement.

### 3.2 Prognostic value of imaging findings

Median OS of the entire sample was 17.3 months (95% CI: 10.6–23.9). The six patients with undetectable LM by FDG‐PET/CT showed a favorable outcome with a median OS of 58 months. The HR for FDG SUVmax in LM, when used as a continuous variable, was estimated to be 1.068; that indicates that a 6.8% increase in hazard of death is expected for each unit increase in SUVmax (95% CI for HR: 1.02–1.10; *p* = 0.001). In a similar vein, the HR for FDG‐ratio, when used as a continuous variable, was estimated to be 1.13; That is, a 13% increase in hazard of death is expected for each unit increase in FDG‐ratio (95% CI for HR: 1.03–1.25; *p =* 0.007).

**FIGURE 3:**
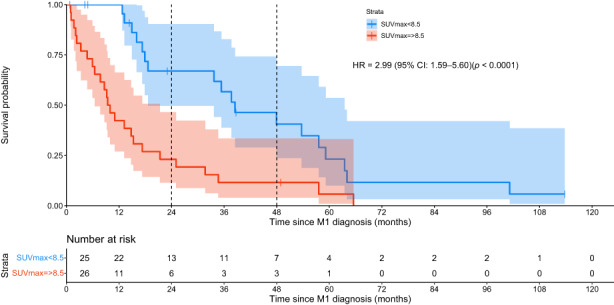
Kaplan–Meier survival curves: FDG‐PET/CT SUVmax—survival curves for 25 patients with low (<8.5) and 26 patients with high (≥8.5) SUVmax. Vertical dotted lines mark the timeline of 2 and 4‐years in both graphs. HR, hazard ratio; M1, metastasis; SUV, standard uptake value.

The observed medians for SUVmax and FDG‐ratio were 8.5 and 1.86, respectively. Kaplan–Meier OS curves for SUVmax <8.5 versus SUVmax ≥8.5, and FDG‐ratio <1.86 versus ≥1.86 are shown in Figure 3 and Figure S2. In the 25 patients whose metastases had a SUVmax <8.5, the median survival was 38.4 months (95% CI: 21.4–55.5). By comparison, in the 26 patients whose metastases had a SUVmax ≥8.5, the median survival was only 9.4 months (95% CI: 6.4–12.3; HR = 2.99; 95% CI for HR: 1.59–5.60; *p* < 0.0001). Interestingly, there were no deaths during the first year after diagnosis of metastatic disease in the group of patients with LM and SUVmax <8.5. These survival differences were maintained over time as median survival at 2 and 4 years were 68% and 35%, respectively, for SUVmax <8.5 patients versus only 23% and 11%, respectively, for patients with SUVmax ≥8.5. Similarly, in the patients whose metastases had a FDG‐ratio <1.86, the median survival was 38.49 months (95% CI: 0.95–79.5), significantly higher than those whose metastases had a FDG‐ratio ≥ 1.86 where the median survival was only 13.19 months (95% CI: 3.9–22.5; HR = 2.39; 95% CI for HR: 1.28–4.48; *p* < 0.006). These survival differences were also maintained over time as median survival at 2 and 4 years were 54% and 36%, respectively, for FDG‐ratio <1.86 patients versus only 29% and 8%, respectively, for patients with FDG‐ratio ≥ 1.86.

Other well‐known prognostic factors for MUM were analyzed. In the univariate analysis, only the presence of extrahepatic disease, diameter of larger LM, SUVmax, and FDG‐ratio retained significance as a continuous or dichotomous variable (Table S3). Both SUVmax and FDG‐ratio were highly correlated, but SUVmax showed the greatest negative impact on patient survival. We found a strong correlation between FDG‐uptake in normal liver with SUVmax and tumor size in M1a disease (Figure S3) that might explain why FDG‐ratio performed worse than SUVmax in predicting OS. In the multivariate analysis, among other prognostic factors, SUVmax remained an independent prognostic factor both when used as a continuous (*p* = 0.011; HR = 1.054; 95% CI: 1.012–1.098) and dichotomous variable (*p* < 0.006; HR = 2.61; 95% CI: 1.320–5.140; Table 2). Diameter of the largest metastasis was the only other prognostic factor that was significant in both models, although other prognostic factors remained significant in the dichotomous model albeit with less significance.

**TABLE 2:** Results of the multivariate survival analysis for the whole population, and for M1a disease.

Variable	Hazard ratio (95% CI)	*p*
Multivariate analysis for all patients (*N* = 51)		
Continuous model		
SUVmax	1.054 (1.012–1.098)	0.011
Diameter of largest metastasis	1.021 (1.012–1.029)	<0.001
Dichotomous model		
GGT (greater than ULN)	2.97 (1.33–6.65)	0.008
SUVmax (≥ median)	2.61 (1.32–5.15)	0.006
Diameter of largest metastasis (≥30 mm)	5.12 (2.19–11.99)	<0.001
Multivariate analysis for M1a patients (*N* = 35)		
Continuous model		
SUVmax	1.081 (1.011–1.156)	0.024
Dichotomous model		
Sex (male)	3.23 (1.05–6.24)	0.014
SUVmax (≥median)	3.08 (1.31–7.30)	0.010

*Note*: Median SUVmax for the whole population was 8.5, and for M1a disease was 6.5.

Abbreviations: GGT, gamma‐glutamyltransferase; ULN, upper limit of normal.

### 3.3 Prognostic value of SUVmax in M1a tumors

**FIGURE 4:**
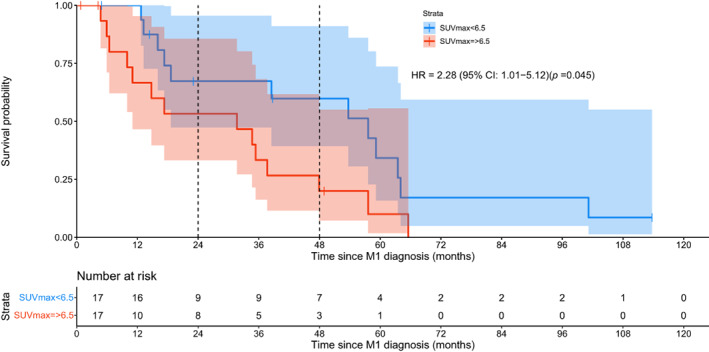
Kaplan–Meier survival curves for patients with M1a disease: FDG‐PET/CT SUVmax—survival curves for 17 patients with low (<6.5) and 17 patients with high (≥6.5) SUVmax. Vertical dotted lines mark the timeline of 2 and 4 years in both graphs. HR, hazard ratio; M1, metastasis; SUV, standard uptake value.

We next wanted to test the value of SUVmax in patients with M1a disease defined as ≤30 mm in the longest diameter of the largest metastasis. Median OS of the 35 patients with M1a disease was 35.3 months (95% CI: 26.7–43.9). The HR for SUVmax in LM, when used as a continuous variable, was estimated to be 1.084. That indicates an 8.4% increase in hazard of death expected for each unit increase in SUVmax (95% CI for HR: 1.009–1.164; *p* = 0.027). The observed median for SUVmax was 6.5 (2.6–22.3). Kaplan–Meier curves for SUVmax <6.5 against ≥6.5 are shown in Figure 4. In the 17 patients whose metastases had a SUVmax <6.5, the median survival was 57.59 months (95% CI: 27.5–87.6). By comparison, in the 17 patients whose metastases had a SUVmax ≥6.5, the median survival was 31.68 months (95% CI: 6.5–58.8; HR = 2.28; 95% CI for HR: 1.01–5.12; *p* = 0.045). Survival differences were maintained over time as median survival at 2 years and 4 years were 64.3% and 53.8%, respectively, for SUVmax <6.5 patients versus 53.3% and 20%, respectively, for patients with SUVmax ≥6.5.

When SUVmax was used in a multivariate analysis among other potential prognostic factors for MUM, SUVmax retained its significance when applying a continuous model (*p* = 0.011; HR = 1.054; 95% CI: 1.012–1.098) and dichotomous model (*p* < 0.0001; HR = 4.072; 95% CI: 1.902–8.716; Table 2). No other prognostic factors were significant in both models, including tumor size as a continuous variable.

## 4 DISCUSSION

To explore the clinical value of the metabolic activity of LM as a prognostic marker in UM, we have collected radiologic and clinical data from a cohort of patients with newly diagnosed MUM treated at a single institute. This report summarizes the analysis of the largest cohort of patients with FDG‐PET/CT in LM from UM. In contrast to the previous smaller studies, this is the only one evaluating prognostic impact of metabolic activity in LM. We were able to find that high metabolic activity measured by FDG‐PET/CT in LM is associated with poor OS. Moreover, high glucose metabolic activity remained significant as an independent variable for OS as a continuous and dichotomous variable.

There are a few small studies in MUM of which most analyze the sensitivity, specificity, and predictive values of FDG‐PET/CT to detect LM compared to morphological imaging with liver MRI.^12^, ^22^ In all of these studies, MRI shows superiority to FDG‐PET/CT for staging of LM from UM. In one study Orcurto et al. identified a total of 108 liver lesions in 10 patients by either MRI and/or FDG‐PET/CT.^22^ Up to 34 lesions (31%) were seen on both modalities, 70 (65%) by MRI only, and only 4 (4%) by FDG‐PET/CT only. FDG‐PET/CT detected 26 of 33 (79%) MRI lesions of ≥12 mm, whereas it detected only 8 of 71 (11%) lesions <12 mm. Our cohort showed the same trend with 90% of lesions ≥12 mm, and 25% of lesions <12 mm being detected by FDG‐PET/CT. The fact that 10–21% of lesions ≥12 mm are not detected by FDG‐PET/CT implies differences in glucose uptake that might not be completely dependent on tumor volume. This difference in glucose uptake is not observed in cutaneous melanoma.^23^ Strobel et al. performed a retrospective evaluation in LM from UM compared to that from cutaneous melanoma. Of 27 LM in 6 of 13 patients (46%) with UM, 16 (59%) were FDG negative, whereas all 43 LM from 14 patients with cutaneous melanoma were positive. LM from UM showed significantly lower SUVmax (mean: 3.5, range: 1.5–13.4) compared with LM from cutaneous melanoma (mean: 6.6, range: 2.3–15.3). Again, all FDG‐PET/CT‐negative LM were detectable by morphological imaging by CT or MRI. In our study, almost 20% of patients presented with iso‐metabolic lesions on the FDG‐PET/CT (FDG‐ratio < 1.1). These differences could be related to different definitions of negative FDG‐PET/CT and different patient populations included. In Strobel's study, more than 50% of patients were alive during follow‐up and mean OS was 26.8 months, so FDG‐PET/CT was probably performed as an extension study in patients that were candidates for liver‐directed therapy. In our study, FDG‐PET/CT was performed as an extension study for patients with newly diagnosed MUM before starting any treatment, including systemic therapy for unresectable disease. The observation that 20%–65% of LM do not show significant glucose uptake increase in the aforementioned studies and our cohort, a phenomenon not observed in cutaneous melanoma, led us to hypothesize that glucose uptake measured by FDG‐PET/CT could be associated with disease prognosis.

The prognostic value of SUVmax in LM has been explored mainly in colorectal cancer. The association was unclear due to heterogeneity between studies and small sample size cohorts until the publication of a meta‐analysis including data from 15 colorectal cancer studies, including 867 patients.^24^ In this meta‐analysis, Xia et al. found that pre‐treatment LM SUVmax was significantly associated with poorer OS with a HR = 1.24 (95% CI for HR: 1.06–1.45) when comparing a low SUVmax group with a high SUVmax group. In our analysis, we also found a negative correlation between LM SUVmax and OS with a HR = 2.99 (95% CI for HR: 1.59–5.60). This HR for MUM is higher than any individual study included in the aforementioned meta‐analysis performed in colorectal cancer LM thus, pointing to a strong association between glucose metabolism and poor prognosis in LM in MUM.

Primary tumor characteristics such as ciliary body involvement, extrascleral extension, diameter and location of the anterior margin of the tumor, cell type, presence of mitotic figures, tumor‐infiltrating lymphocytes and macrophages or the presence of vascular loop, and pigmentation are not prognostic once metastasis has developed.^14^ In addition, it remains unclear if well‐established molecular factors such as chromosome imbalances are prognostic once metastatic disease has been diagnosed. In our experience, Tumor Cancer Genome Atlas (TCGA) molecular classification is not prognostic once LM disease is diagnosed. We have presented results from another cohort of 42 patients with newly diagnosed MUM.^25^ We identified 9 patients with chromosome 3 disomy (D3), and 33 harboring chromosome 3 monosomy (M3) with 22 of them also showing 8q amplification (+8q). Median OS was 14.3 months for D3, 19.1 months for M3, and 10.7 months for M3; +8q patients. Although there is a tendency for shorter OS for M3; +8q patients, the differences are not significant (*p* = 0.12). We were not able to investigate the impact of these prognostic factors in this study because a complete genetic profile was only obtained in 11 patients due to lack of quality and/or quantity of material available in the pathology archive. The median OS for our FDG‐PET/CT cohort of patients with SUVmax <8.5 was 9.4 months, similar to the median OS observed in M3;+8q patients. But the fact that we have representation from all three molecular groups in the 11 patients we have tested makes it unlikely that differences in glucose metabolism is completely associated with classical molecular alterations observed in UM.

With the advent of new therapies to treat MUM patients, there is a renewed need to identify prognostic factors that allow physicians to stratify patients. Until now, only the diameter of the largest metastasis has been included in the AJCC TNM classification system. In this study, we also confirmed the role of the largest tumor M1 as an independent prognostic factor for OS. However, SUVmax was also independently associated with OS as a continuous or dichotomous variable in the whole population, and was the only variable found to be significant as a continuous or dichotomous variable in M1a disease. Interestingly, tumor size was not significant as a continuous variable in M1a disease implying that SUVmax could be a prognostic factor for patients with LM that are ≤30 mm in the longest diameter.

This report has all the limitations inherent in a retrospective study and is only hypothesis generating. Although our report represents the largest analysis in this patient population, the total number of patients remains small. Without prospective studies, no definite recommendations as to the optimal management of LM based on SUVmax can be made. To address that, we have recently initiated a prospective study to evaluate the role of FDG‐PET/TC in patients with newly diagnosed M1a disease. Because UM is a rare disease, we foresee a relatively lengthy recruitment period. In this prospective study, we also plan to address another limitation, the lack of molecular information that allows us to understand the biology behind differences in glucose uptake. A recent paper using data from the TCGA datasets has identified UM as having one of the highest median oxidative phosphorylation (OxPhos) levels among all solid tumors included.^5^, ^6^, ^26^ Using UM cell lines, the authors identified a critical metabolic program dependent on succinate dehydrogenase A (SDHA), the enzyme that couples the tricarboxylic acid cycle with OxPhos by oxidizing succinate. SDHA‐high tumors have elevated expression of OxPhos molecules but are resistant to OxPhos antagonism which can be reversed by SDHA knockdown, thus opening the door to future development of drugs targeting this pathway in MUM.

Despite its limitations, the results of this retrospective study challenge the clinical relevance of FDG‐PET/CT in patients with MUM. FDG‐PET/CT, besides staging information, may provide relevant biologic and prognostic data in the metastatic setting. Interestingly, the prognostic value of FDG‐PET/CT is maintained in M1a tumors. Given its rarity and complexity due to the appearance of treatment options,^7^ patients diagnosed with UM should be referred to centers with expertise with the tumor.^27^

## AUTHOR CONTRIBUTIONS

**Luis P. del Carpio:** Data curation (equal); formal analysis (equal); investigation (supporting); methodology (supporting); writing – original draft (supporting); writing – review and editing (equal). **María Asunción Algarra:** Data curation (equal); formal analysis (equal); investigation (supporting); methodology (supporting); writing – original draft (supporting); writing – review and editing (equal). **Aida Sabaté‐Llobera:** Data curation (equal); formal analysis (equal); investigation (supporting); methodology (supporting); writing – original draft (supporting); writing – review and editing (equal). **Alejo Rodriguez‐Vida:** Data curation (supporting); formal analysis (supporting); investigation (supporting); methodology (supporting); writing – original draft (supporting); writing – review and editing (equal). **Susana Rossi‐Seoane:** Data curation (supporting); formal analysis (supporting); writing – review and editing (equal). **Sandra Ruiz:** Data curation (supporting); formal analysis (supporting); resources (equal); writing – review and editing (equal). **David Leiva:** Data curation (supporting); formal analysis (supporting); investigation (supporting); resources (equal). **Emilio Ramos:** Data curation (supporting); formal analysis (supporting); investigation (supporting); resources (equal); writing – review and editing (equal). **Laura Lladò:** Data curation (supporting); formal analysis (supporting); investigation (supporting); resources (equal); writing – review and editing (equal). **Daniel Lorenzo:** Data curation (supporting); formal analysis (supporting); investigation (supporting); resources (equal); writing – review and editing (equal). **Cristina Gutierrez:** Data curation (supporting); formal analysis (supporting); investigation (supporting); resources (equal); writing – review and editing (equal). **Montserrat Cortes‐Romera:** Data curation (supporting); formal analysis (supporting); investigation (supporting); resources (equal); writing – review and editing (equal). **Josep M. Caminal:** Data curation (supporting); formal analysis (supporting); investigation (supporting); resources (equal); writing – review and editing (equal). **Josep M. Piulats:** Conceptualization (lead); data curation (equal); formal analysis (equal); funding acquisition (lead); investigation (lead); methodology (lead); project administration (lead); resources (equal); software (lead); supervision (lead); validation (lead); visualization (lead); writing – original draft (lead); writing – review and editing (lead).

## FUNDING INFORMATION

The study was conceived and designed by Josep M Piulats who has an intensification grant by the Spanish Society for Medical Oncology (SEOM) through the “Intensificación para investigadores 2020” call. Luis P del Carpio collected the data from clinical reports and performed the analysis. His salary is provided by the Spanish Association Against Cancer (AECC) through the call “Convocatoria de Ayudas Fundación Científica de la AECC A Clínico Junior AECC (convocatoria 2020).” Part of the work such as software license, statistical analyses, and travel expenses have been covered through “Beca SEOM‐JANSSEN para Proyectos de Investigación de Oncología Médica relacionados con Medicina Nuclear 2021” and “I Beca GEM al mejor proyecto GEM.”

## CONFLICT OF INTEREST STATEMENT

Josep M Piulats certifies that all conflict of interest, relationships, and affiliations relevant to the subject matter or materials discussed in the manuscript are the following: Josep M Piulats has acted in a consulting or advisory role for IMMUNOCORE and Bristol‐Myers Squibb (BMS); and has received grants from BMS. Any other authors have conflict of interest relevant to the subject matter or materials discussed in the manuscript.

## ETHICS STATEMENT

All of the methods were conducted in conformity with the Declaration of Helsinki and relevant guidelines. The study was exempted from written informed consent and required ethics approval from the Institutional Review Board of Hospital de Bellvitge/Catalan Cancer Institute at IDIBELL because it was a retrospective study.

## Supporting information


Data S1

## Data Availability

Not applicable.
