# Supplementary material for: Differences in glucose metabolic activity in liver metastasis separates two groups of metastatic uveal melanoma patients with different prognosis
Source: Cancer Med. 2023 May 21;12(13):14062–71. doi: 10.1002/cam4.6058 (PMC10358185; doi:10.1002/cam4.6058)
Supplement: Supplementary file 1 — Data S1 [file CAM4-12-14062-s001.docx]

**SUPPLEMENTARY FIGURES**

**Supplementary Figure 1.-** Large metastases with similar size but completely different metabolic behavior: (A) shows a hypermetabolic lesion in segment II-III of 57 mm diameter with high FDG uptake, while (B) depicts a lesion in segment VI of 60 mm diameter with lower uptake. Both lesions are depicted as hyperintense in T2 (orange arrow) and STIR, and hypointense in T1 with a restricted diffusion pattern by MRI. In both cases the lesions are hipervascular after gadolinium I.V. administration.

**
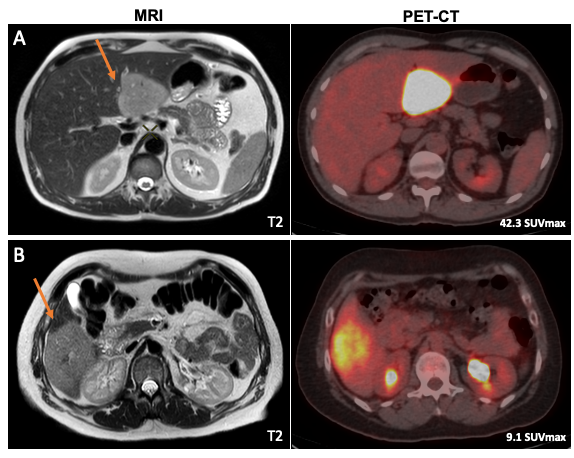
**

**Supplementary Figure 2.-** (A) Kaplan-Meier survival curves: FDG-PET/CT FDG-ratio – survival curves for 25 patients with low (<1.86) and 26 patients with high (≥1.86) SUVmax. Vertical dotted lines mark the timeline of 2-years and 4-years in both graphs.

**
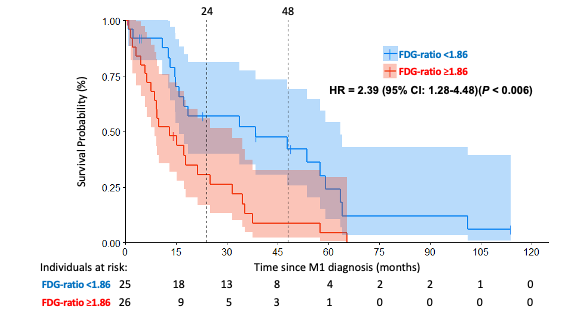
**

**Supplementary Figure 3.-** Linear regression for liver M1 size against normal liver SUV. One model for M1a lesions and another model for lesions M1b-c. A significant positive correlation was observed between size of liver metastases and normal liver SUV for M1a lesions (Beta coefficient = 0.39 (red line), p=0.02). No association was found for larger lesions  (Beta coefficient =0.01 (black line), p=0.9).


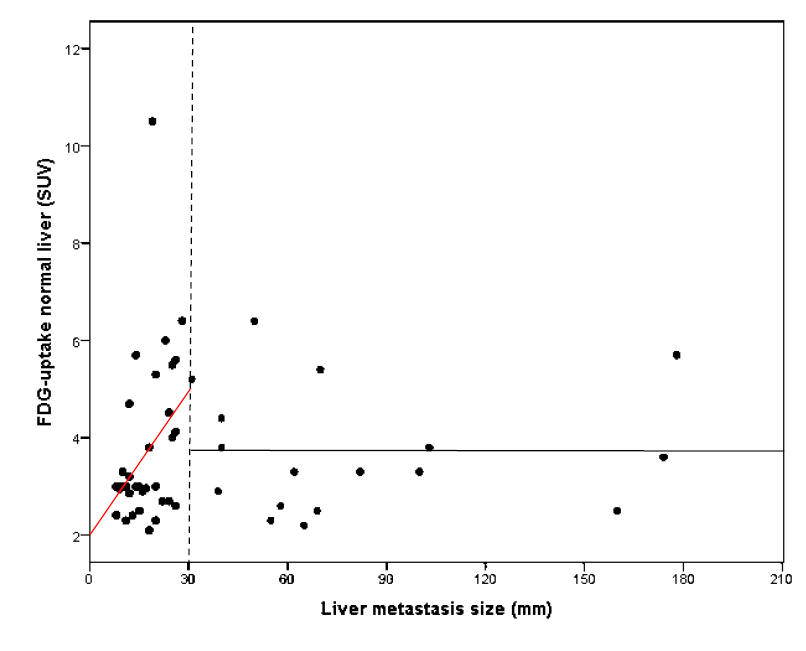


**SUPPLEMENTARY TABLES**

**Supplementary table 1.** Characteristics of the 19 patients with extrahepatic disease. The number represents the SUVmax FDG uptake for the lesion with the highest glucose uptake in each organ affected.

|  |  | **Extrahepatic Disease SUVmax** | | |
| --- | --- | --- | --- | --- |
| **Patient ID** | **Liver M1 SUVmax** | **Bone M1** | **Lung M1** | **Soft Tissue M1** |
| **1** | 3 |  | 2.2 |  |
| **3** | 9.5 |  | 6.5 |  |
| **6** | 20.6 | 12.3 |  |  |
| **7** | 30.2 |  |  | 14.8 |
| **11** | 15.7 | 8 |  | 6.5 |
| **13** | 10.1 | 5.2 | 13.9 | 4.4 |
| **19** | 10.2 |  | 7.9 | 5.3 |
| **21** | 12 |  | 10 |  |
| **24** | 12.8 | 8.8 |  |  |
| **27** | 3.6 | 2.2 |  |  |
| **29** | 3 |  |  | 9 |
| **31** | 11.9 | 3.7 |  |  |
| **32** | 24.2 |  |  | 3.7 |
| **42** | 10.5 |  |  | 3.7 |
| **45** | 6.9 | 2.8 |  |  |
| **50** | 10.2 | 5.3 |  | 3.2 |
| **54** | 6 | 6.3 | 6 |  |
| **58** | 7.2 |  | 2.7 | 9.4 |
| **59** | 8.4 | 6.4 |  |  |
| ID, identification; M1, metastasis. | | | | |

**Supplementary table 2.** Characteristics of patients (data from n=51) with different FDG uptake ratio comparing the lesion with the highest glucose uptake and normal liver uptake.

| **Characteristics** | **Categories** | **Ratio <1.1**  **Number (%)** | **Ratio** ≥**1.1**  **Number (%)** | **p-Value** |
| --- | --- | --- | --- | --- |
|  |  | N=10 (19.6) | N=41 (80.4) |  |
| Sex | Male  Female | 6 (60)  4 (40) | 15 (37)  26 (63) | 0.18 |
| Age, years | < 65 (median 65, range 41-79)  ≥ 65 (median 61, range 30-85) | 6 (60)  4 (40) | 25 (61)  16 (39) | 0.95 |
| ECOG | 0  ≥ 1 | 9 (90)  1 (10) | 31 (76)  10 (24) | 0.32 |
| LDH | Normal  Elevated (greater than ULN) | 6 (60)  4 (40) | 20 (49)  21 (51) | 0.52 |
| ALP | Normal  Elevated (greater than ULN) | 7 (70)  3 (30) | 25 (61)  16 (39) | 0.60 |
| GGT | Normal  Elevated (greater than ULN) | 6 (60)  4 (40) | 16 (39)  25 (61) | 0.32 |
| Site of metastases | Hepatic alone  Hepatic and extra-hepatic | 7 (70)  3 (30) | 25 (61)  16 (39) | 0.60 |
| Diameter of largest metastases  (mm) | < 30  ≥ 30 | 8 (80)  2 (20) | 26 (63)  15 (37) | 0.32 |
| Metastases free survival  (years) | < 2  ≥ 2 | 4 (40)  6 (60) | 14 (34)  27 (66) | 0.73 |
| ECOG, Eastern Cooperative Oncology Group; LDH, lactate dehydrogenase; ALP, alkaline phosphatase; GGT, gamma-glutamyltransferase, ULN, upper limit of normal; * Statistically significant values. | | | | |

**Supplementary Table 3.** Results of the univariate survival analysis.

| **Variable** | **Hazard Ratio (95% CI)** | ***P*** |
| --- | --- | --- |
| ***Univariate analysis***  Sex (male) | 1.56 (0.87-2.99) | 0.18 |
| Age (continuous) | 0.99 (0.96-1.01) | 0.58 |
| ECOG (≥ 1) | 1.06 (0.49-2.31) | 0.87 |
| LDH (greater than ULN) | 1.12 (0.61-2.05) | 0.71 |
| ALP (greater than ULN) | 1.19 (0.64-2.21) | 0.57 |
| GGT (greater than ULN) | 1.86 (0.99-3.94) | 0.06 |
| Metastases free survival (< 2 years) | 1.39 (0.73-2.64) | 0.30 |
| **Site of metastases (extra-hepatic)** | **2.08 (1.08-4.00)** | **0.03*** |
| **Diameter of largest metastasis (continuous)** | **1.02 (1.01-1.30)** | **<0.01*** |
| **Diameter of largest metastasis (≥ 30 mm)** | **5.18 (2.46-10.91)** | **<0.01*** |
| **SUVmax (continuous)** | **1.06 (1.03-1.10)** | **<0.01*** |
| **SUVmax (≥ 8.5)** | **2.99 (1.59-5.65)** | **<0.01*** |
| **FDG-ratio (continuous)** | **1.13 (1.03-1.25)** | **<0.01*** |
| **FDG-ratio (≥ 1.86)** | **1.99 (1.05-3.75)** | **0.03*** |
| ECOG, Eastern Cooperative Oncology Group; LDH, lactate dehydrogenase; ALP, alkaline phosphatase; GGT, gamma-glutamyltransferase, ULN, upper limit of normal; * Statistically significant values. | | |
